# Intravenous Leiomyomatosis in a 20-Year-Old Nulliparous Woman: Atypical Presentation and Radiological Investigation

**DOI:** 10.7759/cureus.90390

**Published:** 2025-08-18

**Authors:** Muhammad Ayub, Sobia Ahmed, Quang Dai La, Eashwar Krishna, Aiman Baloch, Abdul Khaliq, Ayesha Tareen, Pari Gul, Francis Pryor

**Affiliations:** 1 Radiology, Bolan Medical Complex Hospital, Quetta, PAK; 2 Biology, Texas A&M University, College Station, USA; 3 Medicine, The Innovative STEMagazine 501(c)3, College Station, USA; 4 Molecular and Cell Biology, University of Connecticut, Storrs, USA; 5 Medicine, Mekran Medical College, Turbat, PAK; 6 Medicine, Lake Erie College of Osteopathic Medicine, Erie, USA

**Keywords:** cardiac extension, contrast-enhanced ct, gonadal vein, inferior vena cava, intravenous leiomyomatosis, multidisciplinary surgery, pelvic mass, right atrium, uterine fibroid, vascular invasion

## Abstract

Intravenous leiomyomatosis (IVL) is an unusual benign tumor of the uterine smooth muscle, which grows intravascularly. It can spread through the inferior vena cava and into the right heart. We present the case of a 20-year-old female patient who presented with chronic pelvic pain and a background of uterine fibroids. Imaging demonstrated a large pelvic mass with an apparent extension via the right gonadal vein into the inferior vena cava and subsequently into the right atrium and ventricle, consistent with IVL. Despite significant cardiac involvement which resulted in a sizeable mass occupying the pulmonary thoracic cavity, the patient had no evidence of cardiopulmonary symptoms that might be anticipated from the anatomical findings. Diagnosis was made using contrast-enhanced computed tomography. The patient was referred for surgical resection with multidisciplinary support. This case highlights the role of imaging in making the diagnosis of IVL in a timely manner to facilitate surgical management, particularly in younger patients who present in this atypical fashion.

## Introduction

Intravenous leiomyomatosis (IVL) is a rare and unusual condition described in 1896 by Birch‑Hirschfeld and subsequently reported with cardiac extension by Dürck in 1907. It involves histologically benign uterine smooth muscle tumors spreading contiguously into the venous system, occasionally extending to the inferior vena cava (IVC) and sometimes into the chambers of the right heart. IVL is ultimately a benign neoplasm histopathologically; however, the intravascular growth is aggressive and can mimic malignancy [1,2].

The vast majority of cases occur in premenopausal women with a history of uterine fibroids or previous surgery for fibroids, and a strong association with estrogen and progesterone receptor positivity serves to strengthen the opinion that the origin is uterine [3]. Generally, to maintain consistency, there are two dominant pathophysiological theories: first, smooth muscle proliferation and/or initiation within the walls of uterine venular structures, and second, the extension of a primary leiomyoma into the adjacent veins [2].

Clinically, early-stage IVL is often asymptomatic or produces nonspecific pelvic symptoms. As the patient progresses to more advanced disease, some may develop patterns of venous obstruction, remarkable cardiac symptoms, and in some cases, an unexpected intracardiac extension of a tumor causing sudden death [4]. Advances in non-invasive imaging techniques, including CT, MRI and echocardiography, largely facilitate the diagnosis and staging of IVL via the extent of the tumor and its vascular involvement [4]. 

Because it is rare, with only about 400 cases reported thus far, management protocols are often created from single case reports and small series. The mainstay of therapy is surgical removal of the tumor. When necessary, this can be done through a thoracoabdominal excision; however, any oncological resection is the best way to reduce the risk of recurrence. A fair number of recurrences can occur even in cases with incomplete removal of the tumor [1].

This case was presented at the 5th International Radiology Residents Forum (RRF) Conference on October 13th, 2024 at King’s College, London.

## Case presentation

**Figure 1:**
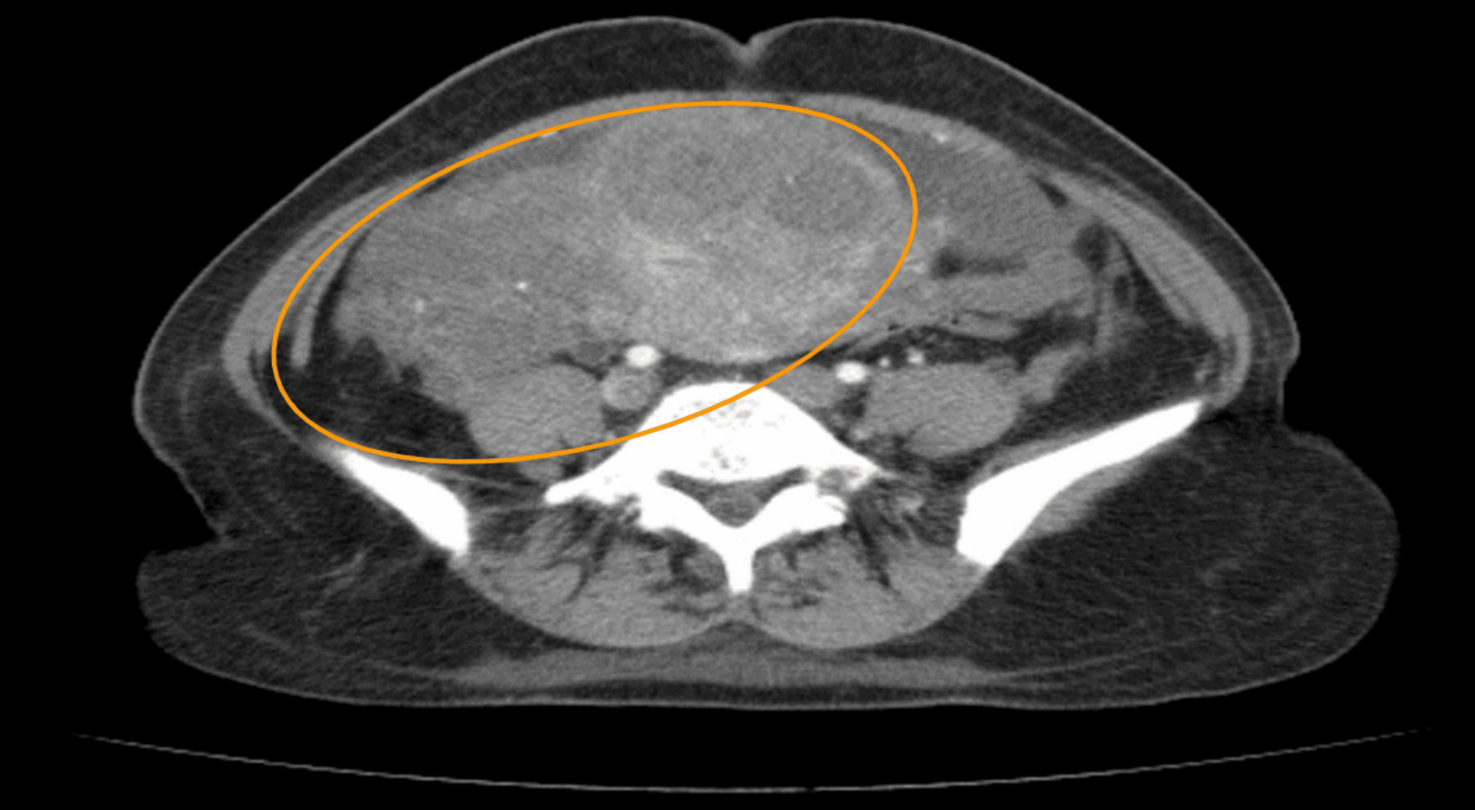
Axial section of contrast-enhanced CT (CECT) in the arterial phase showed a large, lobulated, heterogeneously enhancing soft tissue mass in the pelvis The uterus was not visualized separately from this lesion (indicated by the circle), suggesting either origin from or complete involvement by the mass.

We describe a 20-year-old unmarried female patient who presented to the Bolan Medical Complex Hospital in Quetta, Pakistan, with complaints of chronic pelvic pain and a known history of a large uterine fibroid. An initial ultrasound (US) performed transabdominally highlighted a large pelvic mass, potentially concerning for IVC thrombosis. A contrast-enhanced computed tomography scan (CECT) was recommended to further characterize the mass. Axial views of the CECT demonstrated a large lobulated, heterogeneously enhancing soft tissue mass within the pelvis, and the uterus could not be adequately delineated, suggesting the mass had either originated from or replaced the uterine tissue (Figure 1).

**Figure 2:**
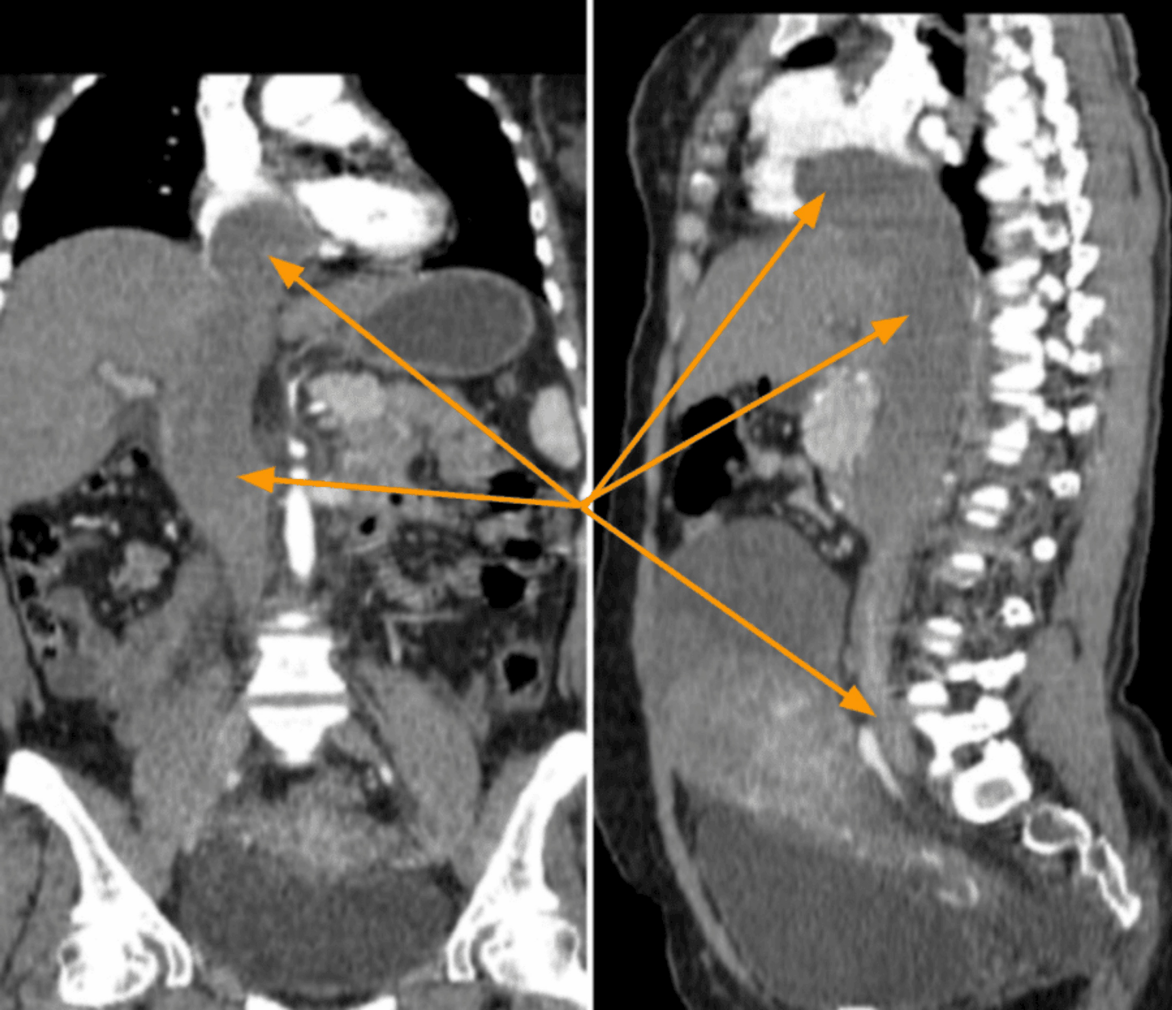
Coronal and sagittal reformatted contrast-enhanced CT images Images showed the pelvic mass invading the right gonadal vein and tracking superiorly into the dilated inferior vena cava (IVC), extending up to the right atrium and protruding into the right ventricle (indicated by the arrows).

Coronal and sagittal reformatted images demonstrated vascular invasion of the lesion extending through the right gonadal vein into the IVC, which appeared markedly dilated. The mass tracked superiorly, encumbering the right atrium, and protruding into the right ventricle (Figure 2).

**Figure 3:**
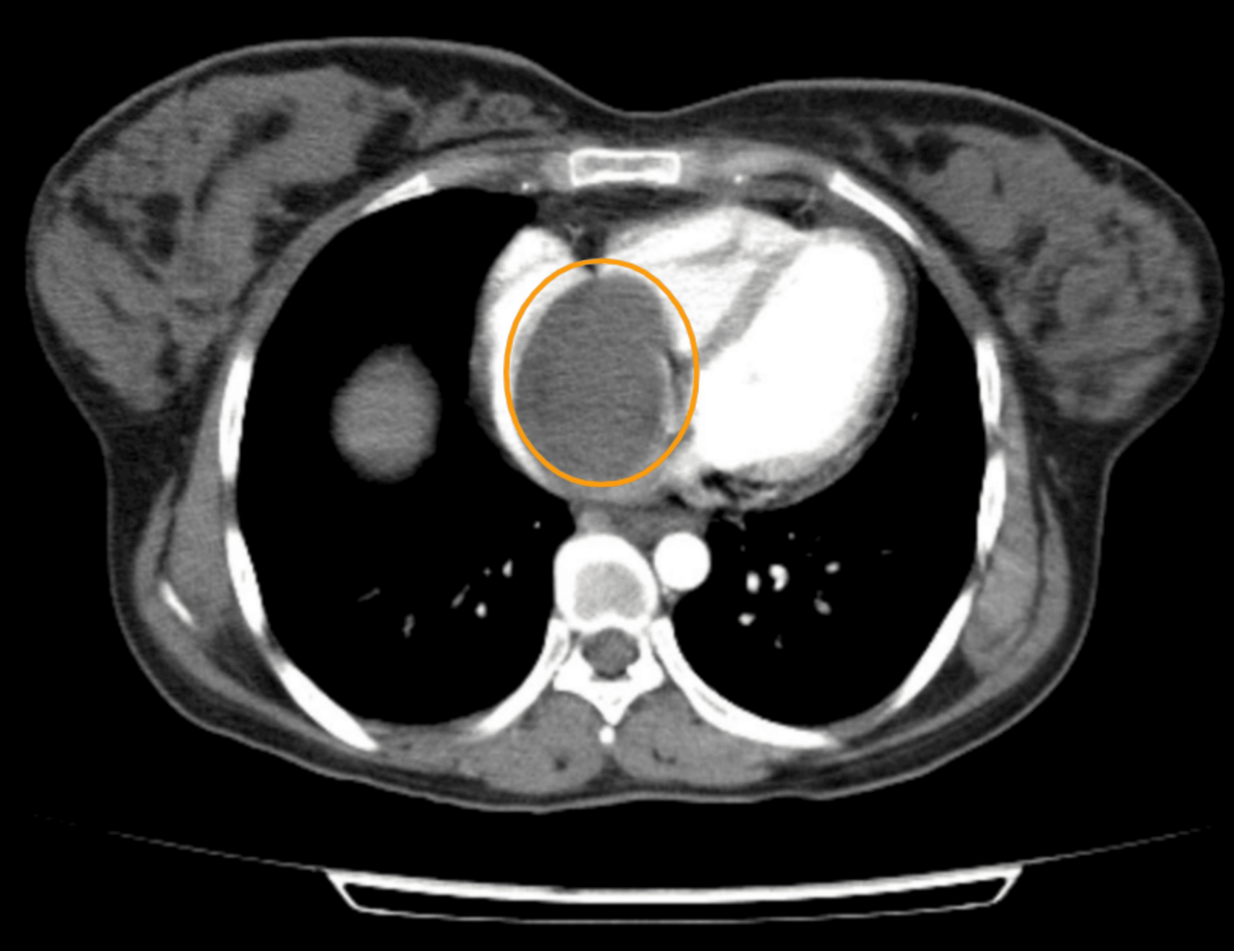
Axial CECT image demonstrated the mass almost completely occupying and distending the right atrium, with further extension into the right ventricle

Subsequent axial sections confirmed that the mass almost completely filled and distended the right atrium and extended into the right ventricle (Figure 3).

For further information, we have also added a video of the CECT below (Video 1).

**Video 1:** Contrast enhanced CT video of the mass

In view of the history of a uterine fibroid and the imaging studies showing the vascular extension of the soft tissue mass into the IVC and right heart, a diagnosis of IVL was made. The patient was referred for assessment via a multidisciplinary team to establish a surgical and cardiothoracic management plan.

The patient was referred to Karachi for management, and we lost contact with the patient's family. The histopathological images are not available because we lost contact with the attendants of the patient, as they moved out of the city for further management. A few days later, the patient passed away.

## Discussion

IVL is a histologically benign smooth muscle tumor that grows with a propensity for an intravascular spread and occasional extension to the right heart. A contemporary review of 672 cases had a mean patient age of 45.5 years, with 35% of patients exhibiting extensions to the renal veins or heart. Preoperative suspicion was present in 55% of patients, and 86% of the cases underwent one-stage surgery. Additionally, 90% of the cases were completely resected, and 12% showed recurrence [5]. This was compatible with the tumor route of our patient; however, our patient's age was markedly different, as she was only 20 years old, which was less than half the average age.

Historically, there are reports dating back to 1989 of IVL invading the vena cava and the heart. Despite being benign histologically, those tumors were positive for estrogen and progesterone receptors [6]. The route via the gonadal veins is well understood in the literature. In the Swedish case from 1989, uterine lesions had invaded the vena cava and atrium, consistent with the route taken in the current patient [6].

A 2021 case report documented a successful excision of an atrial mass in IVL with no recurrence on follow-up. This supports the idea that even if there is an asymptomatic cardiac extension, aggressive surgical intervention is appropriate [2]. While the majority of patients will develop relevant cardiac symptoms to motivate treatment, asymptomatic cases were present in 21% of patients in the case series of 672 patients. This was in line with the silent presentation shown here, even with the extensive cardiac extension [5].

In addition to surgical resection, management strategies typically involve adjuvant hormonal therapy targeting the tumor's estrogen and/or progesterone receptor positivity. Agents such as gonadotropin-releasing hormone agonists (GnRHa), tamoxifen, medroxyprogesterone, or letrozole (an aromatase inhibitor) are used to diminish the growth of the residual tumor or disease recurrence, particularly in cases with margin-positive resection or inoperable disease [2,7,8].

Evidence about the efficacy of postoperative hormonal (anti-estrogen) therapy in reducing recurrence is conflicting: some study results demonstrate stabilization of residual disease or delay in progression with agents like letrozole or GnRHa, when re-resection is not possible, whereas other studies do not show a significant reduction in the risk of recurrence [2,7,9,10].

Long-term follow-up is important for recognizing recurrence, which can vary significantly (10%-30% depending on the completeness of the initial resection and the extent of vascular involvement) [2,10,11], and recommended follow-up typically includes imaging (e.g., pelvic ultrasound, CT, MRI, or venography) at three-, six-, and 12-month intervals postoperatively, with annual imaging afterward. Some experts recommend continued imaging every year for at least 10 years [2,8,12].

The recurrence risk is especially high in patients with residual disease after surgery, especially if the great vessels are involved or if the tumor was incompletely resected, whereas patients with complete excision (i.e., hysterectomy with bilateral salpingo-oophorectomy) have very low rates of relapse [10,11,13,14].

## Conclusions

This case shows the pivotal diagnostic role of imaging in identifying IVL with cardiac extension, particularly in asymptomatic patients. The atypical presentation in a 20-year-old female patient without previous uterine surgery emphasizes the need for increased clinical suspicion for venous involvement in young patients with large uterine masses. Prompt identification and adequate imaging are essential for preoperative planning as complete surgical resection remains the mainstay of treatment. Team-based approaches are essential to minimize the risk of recurrence and enhance long-term success.
